# The hidden risk of ionizing radiation in the operating room: a survey among 258 orthopaedic surgeons in Brazil

**DOI:** 10.1186/s13037-020-00238-6

**Published:** 2020-04-22

**Authors:** Robinson Esteves Pires, Igor Guedes Nogueira Reis, Ângelo Ribeiro Vaz de Faria, Vincenzo Giordano, Pedro José Labronici, William Dias Belangero

**Affiliations:** 1 Departamento do Aparelho Locomotor, Universidade Federal de Minas Gerais, Av. Prof. Alfredo Balena, 190, Santa Efegênia, Belo Horizonte, MG Brazil; 2 Serviço de Ortopedia e Traumatologia Prof. Nova Monteiro, Hospital Municipal Miguel Couto, Rio de Janeiro, RJ Brazil; 3 Departamento de Ortopedia e Traumatologia, Universidade Federal Fluminense, Niterói, RJ Brazil; 4 Departamento de Ortopedia e Traumatologia, Universidade Estadual de Campinas (Unicamp), Campinas, SP Brazil

**Keywords:** Radiation exposure, Ionizing radiation, Radiation, Orthopedics, Scatter radiation, Radiation protection, Occupational exposure, Orthopaedic surgeon

## Abstract

**Background:**

This study aims to assess orthopaedic surgeon knowledge in Brazil about ionizing radiation and its health implications on surgical teams and patients.

**Methods:**

A 15-question survey on theoretical and practical concepts of ionizing radiation was administered during the 23rd Brazilian Orthopaedic Trauma Association annual meeting. The survey addressed issues within orthopedic surgery, such as radiation safety concepts, protection, exposure, as well as the participant gender. Participants were either orthopedic surgeons or orthopedic surgery residents working at institutions in Brazil.

**Results:**

One thousand surveys were distributed at the moment of the meeting registration, and 258 were answered completely (25.8% response rate). Only 5.8% of participants used basic radiation protection equipment; 47.3% used a dosimeter; 2.7% reached the annual maximum permissible radiation dose; 10.5% knew the period of increased risk to fetal gestation; 5.8% knew the maximum permissible radiation dose during pregnancy; 58.5% knew that the hands, eyes, and thyroid are the most exposed areas and at greater risk of radiation-related lesions; 25.2% knew the safe distance from a radiation-emitting tube is 3 m or more; 44.2% knew the safest positioning of the radiation-emitting tube; 25.2% knew that smaller tubes emit greater radiation at the entrance dose to magnify the image; and 55.4% knew that the surgery team receives more scattered radiation in surgical procedures performed on obese patients.

**Conclusion:**

This study revealed inadequate theoretical and practical knowledge about radiation exposure among orthopaedic surgeons in Brazil. Only a minority of orthopaedic surgeons used basic radiation protection equipment. No significant differences in knowledge were found when comparing all orthopedic surgery specialties. Our findings indicate an urgent need for education to increase knowledge among orthopaedic surgeons about the hazards of ionizing radiation. Personal protection and implementation of the ALARA (as low as reasonably achievable) protocol in daily practice are important behaviors to prevent the harmful effects of ionizing radiation.

## Background

In the last few decades, the number of surgeries, new technologies, and diagnostic tools has been increasing along with awareness of radiation exposure risks. In the United States, medical radiation exposure has increased 600% since 1980, and it is estimated that about 2–3% of future cancers could be related to previous ionizing radiation exposure [1]. Assessing what professionals know and do to protect themselves and their patients is important to prevent undesired outcomes while using the latest and best technologies.

Although the harmful effects of radiation on human biology are very well known, the literature contains conflicting evidence concerning some effects of ionizing radiation on professionals [2–4]. Despite awareness of long-term radiation effects, health professionals may still neglect aspects of radiation protection in their daily work.

Fluoroscopy is one of the most valuable tools in the orthopaedic surgeon arsenal, especially for fracture reduction and proper implant placement. Correct C-arm operation via clear communication with the technician is crucial to obtain useful images. Furthermore, orthopaedic surgeons should aim to keep radiation exposure “as low as reasonably achievable” (ALARA), to decrease risks to both patient and staff [2–6].

The aim of this study is to assess the knowledge of orthopedic surgeons in Brazil on ionizing radiation and protection recommendations, broken down by specialty. The study also evaluates some implications regarding the health of the surgeon, surgical team, and patients and includes some controversial topics found in the literature.

## Methods

**Fig.1:**
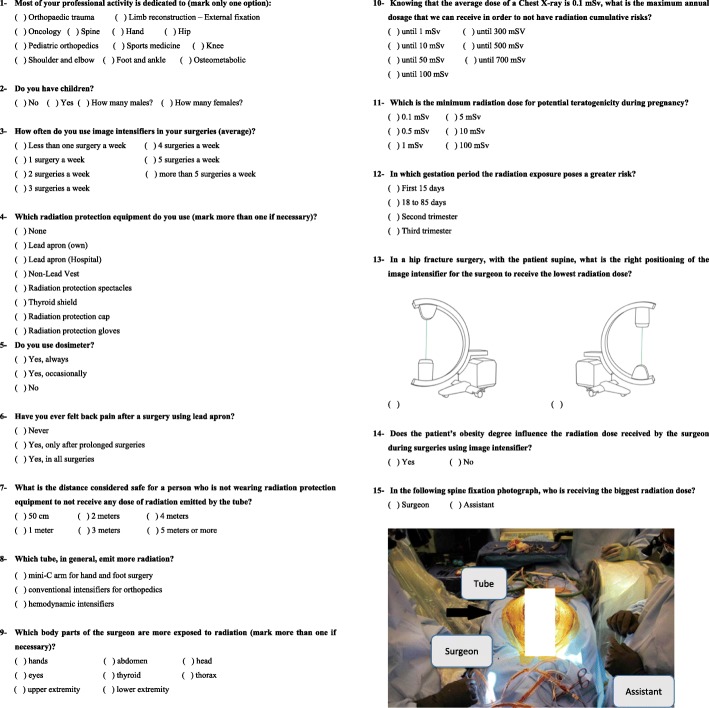
**a** and **b**: Survey containing theoretical and practical concepts about ionizing radiation

During the 23rd Brazilian Orthopaedic Trauma Association annual meeting, we distributed 1000 surveys at the moment of the attendees registration, containing 15 questions (Fig. 1a and b) regarding theoretical and practical concepts of ionizing radiation. Inclusion criteria were being an orthopaedic surgeon or orthopaedic surgery resident at an institution in Brazil. In total, 258 surveys were completed.

Contingency tables were used for data description. Categorical variables were tested by Chi-square test, and results were considered significant when *p* < 0.05. Spearman correlation test was also used for continuous/categorical variables against another categorical variable (more than 2 categories each), considering a confidence interval (CI) of 95% and significance of 5%.

## Results

Table 1 shows the distribution of protection equipment use according to equipment type and number of orthopaedic surgeons in the study. Among 258 participants, 256 (99.2%) used some kind of radiation protection, and 2 (0.8%) used none. Additionally, 170 (65.9%) only used the apron as protection, and 68 (26.3%) used the apron and thyroid shield. Only 5.8% used the apron, thyroid shield, and radiation protection glasses (Table 1).

**Table 1:** Types and frequency of protection equipment use

Protection Equipment	Frequency	%
Hospital Apron	169	65.5
Hospital Apron + Thyroid shield	67	25.9
Hospital Apron + Thyroid shield + Spectacles	13	5.0
Hospital Apron + Spectacles	2	0.8
Own Apron	1	0.4
Own Apron + Thyroid shield	1	0.4
Own Apron + Thyroid shield + Spectacles	2	0.8
Own Apron + Spectacles	1	0.4
None	2	0.8
**Total**	**258**	**100**

Table 2 shows the frequency of radiation protection equipment use by specialty. Orthopaedic trauma surgeons used the most protection equipment, with 47% using two or more items. In contrast, shoulder and pediatric surgeons had the lowest percentages of equipment use, 15 and 0% (≥ two or more items of equipment), respectively. They were also the only groups containing professionals who used no protection at all. According to Chi-square test, there is a statistically significant difference between the groups “no protection equipment used” and “≥ two pieces of equipment used” (Table 2).

**Table 2:** Radiation protection equipment use by specialty

Specialty	Protection Equipment Use (Apron, Thyroid Shield, Spectacles)		
	None	One equipment	≥ Two pieces of equipment use
Knee	0.00	32.00	6.00
Hand	0.00	14.00	3.00
Shoulder	1.00	16.00	3.00
Foot and Ankle	0.00	11.00	4.00
Pediatric	1.00	5.00	0.00
Hip	0.00	21.00	7.00
Trauma	0.00	71.00	63.00
**Total**	**2.00**	**170.00**	**86.00**
***p*-value**	0.000*	**0.287**	0.009*

*statistically significant: *p*-value < 0.05

Table 3 shows the number of surgeries requiring fluoroscopy per week per surgeon and the gender distribution of surgeon offspring. Regarding radiation exposure affecting surgeon offspring, no statistically significant result was identified (Tables 3 and 4). No significant difference in offspring gender was apparent when analyzing each radiation exposure group (Table 3).

**Table 3:** Distribution of fluoroscopy use frequency and offspring sex

Surgeries that requires fluoroscopy per week per surgeon	Number of Participants	Offspring sex		***p***-value
		Male	Female	
Less than 1 surgery	7	4	1	0.19
1 surgery	25	6	6	0.96
2 surgeries	44	23	21	0.83
3 surgeries	54	26	18	0.27
4 surgeries	66	29	32	0.62
5 surgeries	35	11	16	0.62
More than 5	27	10	12	0.30
**Total**	258	109	106	

*statistically significant: *p*-value < 0.05

**Table 4:** Fluoroscopy use and male proportions in offspring of male orthopaedic surgeons

Groups	N° of offspring	N° of male births	Male proportion (%)	Sex Ratio	OR	95% CI
Reference population	91,893,674	46,158,225	50,23	1,01	1	Reference
All irradiated groups	215	109	50,70	1,03	1019	0,780 - 1331
Until 2 surgeries per week	61	33	54,10	1,18	1168	0,076 - 1932
3 or more surgeries per week	154	76	49,35	0,97	0,965	0,074 - 1324

Similarly, no significant difference was found when considering orthopaedic surgeons who used equipment up to 2 times per week and those who used it 3 or more times per week (Table 4).

The dosimeter was not used by the majority of the sample, 136 participants (52.7%) and only 22.1% always used it. No significant difference occurred with dosimeter use among all the specialties analyzed (Table 5).

**Table 5:** Comparison between specialty and dosimeter use

Specialty	Dosimeter use		Total	***p***-value
	Yes	No		
Knee	12	26	38	0.283
Hand	11	6	17	
Shoulder	11	9	20	
Foot and Ankle	8	7	15	
Pediatric	2	4	6	
Hip	12	16	28	
Trauma	66	68	134	
Total	122	136	258	

Occurrence of back pain presented no statistically significant difference when analyzing back pain and the use of lead apron (Table 6). Most of the individuals had back pain during long surgeries (82.2%).

**Table 6:** Comparison between back pain and lead apron use

Back pain frequency	Lead apron use				Total	***p***-value
	Yes	%	No	%		
Never	99	38.7	2	100.0	101	0.209
Prolonged surgeries	129	50.4	0	0.0	129	
All surgeries	28	10.9	0	0.0	28	
**Total**	**256**	**100.0**	**2**	**100.0**	**258**	

Regarding the variables of distance, tube plus radiation, exposed body parts, maximum annual dose, gestation maximum dose, and gestation period, no statistically significant difference occurred when comparing the knowledge of each specialist group across the variables mentioned in Table 7 (Table 7).

**Table 7:** Analysis’ results between specialties for each variable

Variable	Correlation Coefficient	***p***-value
Distance	−0.036	0.562
Tube plus Radiation	−0.018	0.777
Exposed body parts	−0.035	0.581
Annual maximum dose	−1.060	0.089
Gestation maximum dose	−0.017	0.790
Gestation period	−0.021	0.737

No differences appeared regarding fluoroscope tube positioning, obesity, and radiation dose scenario. In this study, no individual orthopaedic surgery specialty appeared to have more knowledge about radiation (Table 8).

**Table 8:** Comparison between variables and specialties

Specialty	Fluoroscope tube positioning				Total	***p***-value
	Below	%	Over	%		
Knee	23	16.0	15	13.2	38	0.614
Hand	7	4.9	10	8.8	17	
Shoulder	11	7.6	9	7.9	20	
Foot and Ankle	7	4.9	8	7.0	15	
Pediatric	5	3.5	1	0.9	6	
Hip	17	11.8	11	9.6	28	
Trauma	74	51.4	60	52.6	134	
Total	144	100.0	114	100.0	258	
**Specialty**	**Obesity**				**Total**	***p*-value**
	**Yes**	**%**	**No**	**%**		
Knee	26	18.2	12	10.4	38	0.408
Hand	6	4.2	11	9.6	17	
Shoulder	12	8.4	8	7.0	20	
Foot and Ankle	7	4.9	8	7.0	15	
Pediatric	3	2.1	3	2.6	6	
Hip	15	10.5	13	11.3	28	
Trauma	74	51.7	60	52.2	134	
Total	143	100.0	115	100.0	258	
**Specialty**	**Radiation dose in scenario (Question 15 of questionnaire)**				**Total**	***p*-value**
	**Assistant**	**%**	**Surgeon**	**%**		
Knee	17	11.3	21	19.4	38	0.468
Hand	9	6.0	8	7.4	17	
Shoulder	13	8.7	7	6.5	20	
Foot and Ankle	8	5.3	7	6.5	15	
Pediatric	5	3.3	1	0.9	6	
Hip	16	10.7	12	11.1	28	
Trauma	82	54.7	52	48.1	134	
Total	150	100.0	108	100.0	258	

## Discussion

Since 1980, the United States has experienced a sixfold increase in medical radiation exposure. Estimates suggest that up to 3% of all future malignant neoplasia could be caused by previous ionizing radiation exposure [1]. Several studies, therefore, have been and are being conducted to gather information to develop education for using the best available evidence and technology, while preventing harm to patients and medical teams.

Currently, the literature contains conflicting evidence regarding some effects of ionizing radiation. One interesting idea is that daily radiation exposure in male doctors while working may increase the chances of producing female offspring. Zadeh and Briggs published one of the first related studies in 1997. They reported that male obstetricians, gynecologists, and orthopaedic surgeons in the United Kingdom had a higher incidence of female offspring [7]. In addition, an increased risk for congenital abnormalities was present, and a statistical difference existed in all the findings compared to the population [7]. Since the obstetricians and gynecologists were not exposed to radiation, Zadeh and Briggs proposed that occupational exposure to x-ray was not associated with the findings, and the possible cause was exposure to the operating theatre environment [7]. However, Hama et al. from Japan divided participants of his study into two groups, “lightly irradiated” and “highly irradiated” (one or more incidents of annual radiation exposure > 10 mSv). They found a significant statistical increase in the risk of radiologists from the “highly irradiated” group producing a lower proportion of male offspring [8]. The most recent study on this topic published by Choi et al. used a sample of male invasive cardiologists. The authors found no significant difference in the proportion of male and female offspring, even when analyzing a subgroup with higher radiation exposure [9]. In the present study, we found similar results to Choi et al. and Zadeh and Briggs. No difference was identified in the proportion of male offspring born to male orthopaedic surgeons in Brazil. The proportions were similar to the Brazilian population. We also compared a group of lower radiation exposure to one of higher exposure, determined by the number of surgeries requiring fluoroscopy per week per surgeon. Again, no difference was found in offspring gender proportion.

Another controversial topic is the relationship between lead apron use and back pain in orthopaedic surgeons or professionals that deal with x-rays. To our knowledge, the first study investigating this relationship was published by Moore et al. and did not prove lead apron use as a risk factor for back pain [10]. Later, research on the prevalence of spinal disc disease among interventional cardiologists argued the existence of the “interventionalist’s disc disease”. It reported significant differences between the incidence of skeletal complaints among interventional cardiologists compared to orthopaedic surgeons and rheumatologists [11]. Their study showed a greater incidence of cervical, rather than lumbar, problems. It was also noted that interventional cardiologists use aprons for longer periods, which increases the impact on the axial skeleton [12]. However, our study produced results similar to Moore. No significant relationship occurred between back pain frequency and apron use among orthopaedic surgeons in Brazil, but the descriptive analysis showed that most of our participants complained of back pain during prolonged surgeries. Therefore, we think that more hours of apron use might be necessary to cause harmful effects and related back pain, similar to the study of interventional cardiologists. Even without apron use, prolonged procedures might be a cause of back pain, although we have yet to see a study comparing both situations.

Our study also assessed how orthopaedic surgeons in Brazil protect themselves from occupational ionizing radiation and whether they know the theory behind prevention from harmful x-rays. Unfortunately, the results showed a lack of radiation protection equipment use and lack of knowledge about basic radiation prevention. In our sample, 65.9% only used the apron as protection equipment despite the well-known fact that radiation is the main risk factor for thyroid cancer. Only 32.1% used at least the apron and thyroid shield, and 5.8% used the apron, thyroid shield, and radiation protection glasses. The dosimeter was not used by the majority, 52.7%, and only 22.1% always used it. A study about fluoroscopic radiation exposure highlighted that surgeon eyes and hands receive more radiation than other body parts, and therefore surgeons should routinely use eye and hand protection in addition to the apron and thyroid shield [13]. It is noteworthy that a study published by Muir et al. showed that some aprons were labeled as having higher protection than they in fact presented when tested [14].

Regarding the questions on radiation prevention knowledge, most of the orthopaedic surgeons answered poorly. Only 2.7% reached the acceptable annual maximum permissible radiation dose. Just 10.5% knew the period of greater risk to the fetus when exposed to x-rays, and 5.8% reached the maximum permissible radiation dose during pregnancy. About one quarter, 25.2%, knew that 3 m or more from the radiation-emitting tube is considered the safe distance, and 44.2% knew the safest positioning of the radiation-emitting tube. Only 25.2% knew that smaller tubes generally emit greater radiation at the entrance dose to magnify the image. Just over half, 55.4%, knew that the surgery team receives more scattered radiation in surgical procedures performed on obese patients. Finally, the question if the hand, eyes, and thyroid are the most exposed and at greater risk of radiation-related lesions was correctly answered by only 58.5% of participants.

No significant differences in knowledge were found when comparing all orthopaedic surgery specialties. All specialties performed similarly. It seems that the poor performance on the survey is not limited to Brazil. An original study in 2013 showed that orthopaedic surgeons from Canada lacked knowledge about the risk of eye cataracts when exposed to radiation, and 75% were unaware of radiation dose limits [15]. A survey analysis from Turkey demonstrated inadequate knowledge about the uses and risks of fluoroscopy and radiation prevention [16]. Another article from Latin America showed that 75.7% of their sample rarely or never used a dosimeter badge, and only 20.2% used lead glasses in their practice. The article also highlighted significant differences and many knowledge deficiencies among countries [17].

An interventional study was performed to analyze the effect of surgeon education about radiation protection [18]. The educational intervention was applied to surgeons performing complex endovascular procedures. A strong relationship (*p* < 0.001) between the intervention and decrease in radiation dose was found, excluding cases of fenestrated endovascular aneurysm repair which continued to present high radiation exposure [18]. Additionally, some articles summarize the main important aspects of how to decrease unnecessary radiation exposure, by explaining radiation effects, dose, and protection equipment. These articles highlight that minimally invasive surgery increases radiation exposure, especially in cases of spinal surgery [19, 20]. The concepts of ALARA (as low as reasonably achievable) and DEBT (distance, exposure, barriers, and time) are highlighted as pillars of practical guidelines [2–6, 21].

As a main limitation, our study design and sample characteristics preclude concluding that orthopaedic surgeons worldwide present the same knowledge regarding radiation exposure and safety procedures to avoid harmful effects. Another limitation is the lack of uniformity among subspecialties. We had 134 trauma surgeons versus 6 pediatric surgeons, 15 foot and ankle surgeons, and 17 hand surgeons, potentially limiting comparison among specialists in terms of knowledge and other variables analyzed in the study. Nevertheless, by focusing on basic education for medical residents before specialization, we feel our data demonstrates quite clearly that orthopedic practitioners present a low level of knowledge regarding the harmful effects of radiation exposure.

## Conclusion

Our study reveals that orthopaedic surgeons in Brazil presented inadequate performance regarding theoretical and practical knowledge about radiation exposure. No significant differences were found when comparing knowledge in any topic among all orthopaedic surgery specialties. Furthermore, only the minority of orthopaedic surgeons used the basic radiation protection equipment (apron, thyroid shield, and radiation protection glasses). The data in this study highlight an urgent need to create education for orthopaedic surgeons and orthopaedic surgery residents in Brazil, and possibly worldwide, to decrease patient and surgeon exposure to ionizing radiation. Personal protection and implementation of the ALARA protocol in daily practice are important behaviors to help prevent the harmful effects of ionizing radiation.

## Abbreviations

CI: Confidence Interval
mSv: Milisievert
ALARA: As Low As Reasonably Achievable
DEBT: Distance, Exposure, Barriers, and Time
